# Detergent-Mediated Virus Inactivation in Biotechnological Matrices: More than Just CMC

**DOI:** 10.3390/ijms24097920

**Published:** 2023-04-27

**Authors:** Jean-Baptiste Farcet, Michael Karbiener, Leonhard Zelger, Johanna Kindermann, Thomas R. Kreil

**Affiliations:** 1 Pharmaceutical Sciences, Baxalta Innovations GmbH, Now Part of the Takeda Group of Companies, 1221 Vienna, Austria; jean-baptiste.farcet@takeda.com; 2 Global Pathogen Safety, Takeda Manufacturing Austria AG, 1221 Vienna, Austria; michael.karbiener@takeda.com (M.K.); johanna.kindermann@takeda.com (J.K.)

**Keywords:** virus inactivation, Triton X-100, detergent, Nereid, Triton X-100 reduced, CMC, enveloped virus, plasma product, membrane disruption, S/D treatment

## Abstract

For decades, the ability of detergents to solubilize biological membranes has been utilized in biotechnological manufacturing to disrupt the lipid envelope of potentially contaminating viruses and thus enhance the safety margins of plasma- and cell-derived drugs. This ability has been linked to detergent micelles, which are formed if the concentration of detergent molecules exceeds the critical micelle concentration (CMC). Traditionally, the CMC of detergents is determined in deionized water (ddH_2_O), i.e., a situation considerably different from the actual situation of biotechnological manufacturing. This study compared, for five distinct detergents, the CMC in ddH_2_O side-by-side with two biopharmaceutical process intermediates relevant to plasma-derived (Immunoglobulin) and cell-derived (monoclonal antibody) products, respectively. Depending on the matrix, the CMC of detergents changed by a factor of up to ~4-fold. Further, the CMC in biotechnological matrices did not correlate with antiviral potency, as Triton X-100 (TX-100) and similar detergents had comparatively higher CMCs than polysorbate-based detergents, which are known to be less potent in terms of virus inactivation. Finally, it was demonstrated that TX-100 and similar detergents also have virus-inactivating properties if applied below the CMC. Thus, the presence of detergent micelles might not be an absolute prerequisite for the disruption of virus envelopes.

## 1. Introduction

Human immunodeficiency virus (HIV) and Hepatitis C virus (HCV) were transmitted through plasma-derived medicinal products in the 1980s [1,2]. To minimize any chance of reoccurrence, treatment with a combination of solvent and detergent (S/D), often Triton X-100 (TX-100) and Polysorbate 80 (PS80) with Tri-n-butyl-phosphate (TnBP), has been implemented into the manufacturing of drugs that originate from plasma [3]. As of now, S/D treatment is still considered a very effective and robust method to inactivate lipid-enveloped viruses [4]; it is also used in the manufacturing of cell-derived medicinal products. However, with regard to TX-100, environmental concerns have been raised recently [5], as its metabolites may act as endocrine disruptors once released in the wastewater effluents [6]. Hence, the EU has decided to restrict (from 2021) and ultimately prohibit the use of this chemical [7], which has challenged the pharmaceutical industry to find a suitable surrogate compound [8,9]. Two potential replacement candidates, Triton X-100 reduced (TX-100R) and Nereid (a newly synthetized compound), have been identified by our group as particularly well suited, showing similar structural properties and similar inactivation potency to TX-100, even when applied as a single detergent [10]. However, their mechanism of action remains not fully understood. Potentially, the formation of detergent micelles is essential for virus inactivation, which is dependent on the solution matrix and temperature, but also on the chemical structure of the surfactants [11]. The driving force for micelle generation is the elimination of contact between a detergent’s alkyl chains and the surrounding matrix in order to minimize interactions with water. While a detergent’s critical micelle concentration (CMC, the minimum concentration at which micelles are formed) is routinely determined at ~25 °C in deionized water (ddH_2_O), it is unknown to what extent this value might change under biopharmaceutical manufacturing conditions, i.e., in the presence of salts and proteins and at different temperatures. Further, while Gooran et al. have recently postulated an indispensable role of micelle formation, the corresponding experiments were based on modeling the interaction of detergents with the virus lipid envelope via a supported lipid bilayer platform; thus, they did not involve virus infectivity assays [12]. To shed further light on these topics, the present study employed force tensiometry to determine the CMCs of several detergents in distinct biotechnological process intermediates at different temperatures. The resulting information enabled the subsequent conduction of virus inactivation studies below and above the determined CMCs.

## 2. Results

### 2.1. CMC Determinations

**Figure 1:**
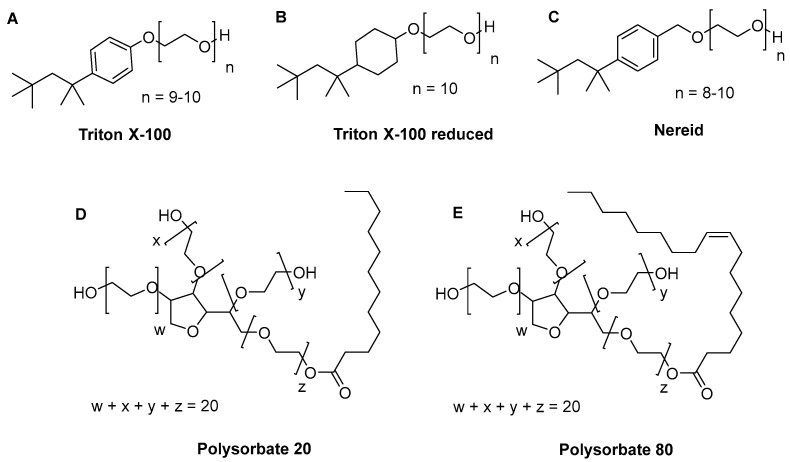
Chemical structure of the investigated detergents (**A**) Triton X-100, (**B**) Triton X-100 reduced, (**C**) Nereid, (**D**) Polysorbate 20, and (**E**) Polysorbate 80.

Preliminary force tensiometry experiments with S/D mixtures (e.g., the frequently applied mixture of TX-100, PS80, and TnBP (all Merck; Rahway, NJ, USA)) revealed ambiguous measurement curves that precluded reliable CMC determination (data not shown). Thus, we focused on single detergent experiments with five distinct compounds (Figure 1) that were previously shown to possess high (TX-100, Nereid (Takeda, Vienna, Austria), TX-100R (St. Louis, MO, USA)) or comparatively lower (PS80, Polysorbate 20 (PS20 (Merck)) antiviral potency [10]. Force tensiometry runs were conducted with each of these detergents in ddH_2_O, as well as in two distinct product intermediates that are relevant for either plasma-derived products (immunoglobulin, the ‘plasma protein matrix’) or cell-derived products (monoclonal antibody, the ‘recombinant protein matrix’). As for the product intermediates, temperatures were chosen to reflect the respective conditions of large-scale manufacturing. For all conditions except the measurement of PS80 in the recombinant protein matrix, raw data were obtained that permitted reliable CMC determination (see Section 4.3 and Figure S1, provided as Supplementary Materials).

**Figure 2:**
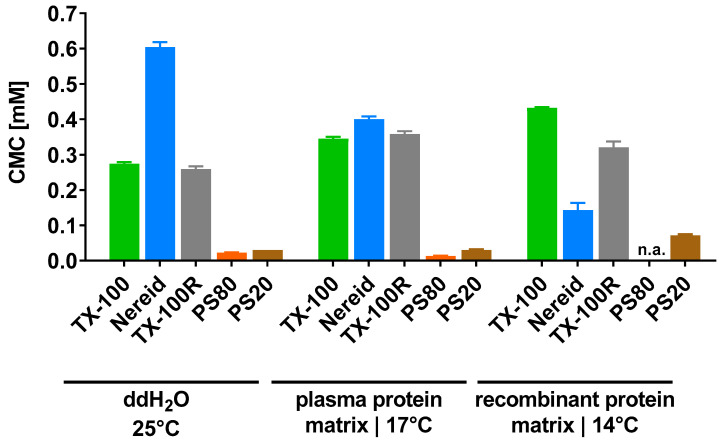
Critical micelle concentrations of the detergents TX-100, Nereid, TX-100R, PS80, and PS20, which were determined by force tensiometry in ddH_2_O (25 °C), a plasma-derived model matrix (17 °C), and a recombinant protein model matrix (14 °C). Data are shown as mean ± SD of three independent determinations, except for PS20 in the plasma protein matrix (two independent determinations). n.a.—not applicable.

The propensity for micelle formation was influenced by the matrix in a non-predictable fashion (Figure 2). For instance, the CMC of TX-100 was higher in the biopharmaceutical product intermediates (plasma protein matrix: 0.345 mM; recombinant protein matrix: 0.432 mM) than in ddH_2_O (0.274 mM), which was also seen for TX-100R (plasma protein matrix: 0.358 mM; recombinant protein matrix: 0.321 mM; ddH_2_O: 0.259 mM); yet, the opposite was observed for Nereid (plasma protein matrix: 0.400 mM; recombinant protein matrix: 0.143 mM; ddH_2_O: 0.604 mM). Compared to these three detergents, markedly lower CMCs were determined for PS80 (plasma protein matrix: 0.013 mM; ddH_2_O: 0.022 mM) and PS20 (plasma protein matrix: 0.030 mM; recombinant protein matrix: 0.071 mM; ddH_2_O: 0.030 mM).

### 2.2. Virus Inactivation below and above Detergent CMCs

**Figure 3:**
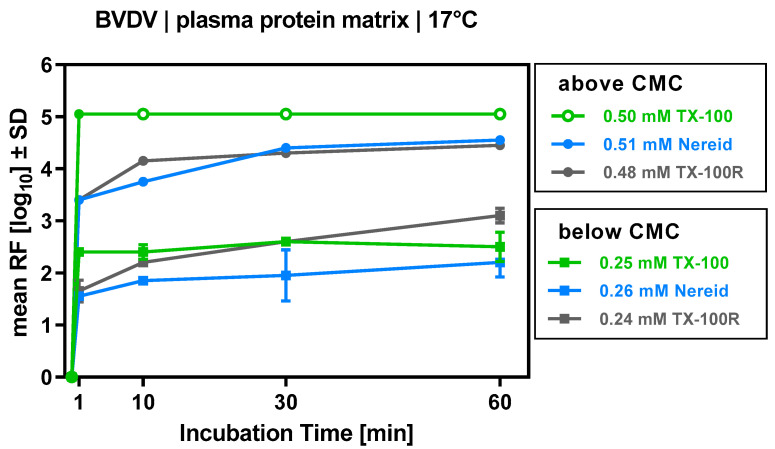
Inactivation of bovine viral diarrhea virus (BVDV) by TX-100, TX-100R, and Nereid employed as single detergents above and below their critical micelle concentration (CMC). Virus clearance was evaluated over a time of 60 min for the plasma-derived model matrix at 17 °C. Virus inactivation performance is depicted as reduction factor (RF), i.e., the log_10_-transformed ratio of (a) viral load before addition of detergent mix and (b) viral load at either 1, 10, 30, or 60 min after addition of detergent. Open circles denote samples for which no residual infectious virus could be detected (assay detection limit was approached). Each data point is the mean of two samples that were drawn from separate experimental runs. Error bars denote SD (only shown if larger than the height of symbols).

For the biotechnological matrices, the preceding CMC determinations had enabled us to pinpoint the ‘separating line’ above which a detergent would form micelles, while below it would not; this was also achieved at the respective virus clearance step at manufacturing scale. With this information to hand, we aimed to study virus inactivation above and below this boundary. To approach the former condition, TX-100, Nereid, and TX-100R were employed at concentrations that translate to only 3% of the target TX-100 concentration in manufacturing (approximately 0.5 mM). Bovine viral diarrhea virus (BVDV; a model for HCV) was effectively inactivated by all three compounds, with comparably fast inactivation kinetics (Figure 3). The final log_10_ reduction factors (RFs) were >6.1 (TX-100; taking into account the volume of all samples where no viral infectivity was detected), 4.6 (Nereid) and 4.5 (TX-100R), respectively. To approach conditions below the CMC, the same detergents were employed at 1.5% of the target TX-100 concentration in manufacturing (approximately 0.25 mM). Compared to the first set of experiments, decreased BVDV inactivation was noted; however, for all three detergents, a reliable and fast inactivation was still evident (Figure 3), with final log_10_ RFs of 2.5 for TX-100, 2.2 for Nereid, and 3.1 for TX-100R.

## 3. Discussion

Detergents have a long-standing tradition as tools in biochemical research due to their ability to interact with biological membranes and thereby solubilize membrane proteins for downstream investigations [13]. Historically, this feature has been linked to the formation of detergent micelles; i.e., the use of a detergent at a concentration above its CMC [14]. The very same ability of detergents to interact with biological structures of lipid membranes and amphiphilic membrane proteins is also the underlying principle of its use in virus inactivation [15]. Indeed, no transmission of enveloped viruses by any plasma-derived S/D-treated product has been reported since the incorporation of this process engineering step (more than 30 years ago) [4], which underscores the high utility and robustness of this safety measure.

However, due to its detrimental effects on the environment, one of the most frequently employed antiviral detergents—TX-100—will soon have to be excluded from industrial processes, at least in the EU. Previous investigations have suggested TX-100R and Nereid to be structurally related, vital alternatives with respect to virus inactivation [10], and have also dissected the influence of TX-100′s different structural domains on its antiviral potency [16]. The present study was designed to compare the micelle formation of these and other detergents, starting from the traditional setting of investigating detergent micelle formation in ddH_2_O at 25 °C. It should be noted that the CMCs obtained for TX-100 and TX-100R in ddH_2_O were in good agreement with previously published results [13,17], supporting the general validity of our experimental setup. Interestingly, the CMC of Nereid in ddH_2_O was ~2-fold higher than the (very similar) values obtained for TX-100 and TX-100R, showing that even subtle changes in chemical structure—a difference of a single methylene group for Nereid vs. TX-100—can have notable effects on micelle formation.

Importantly, the present study also shows that a detergent’s CMC, usually specified for ‘standard conditions’, i.e., in ddH_2_O and at 25 °C, is not the same in a biotechnological matrix and at the respective temperature employed in biotechnological manufacturing. In most instances, we measured fold changes of ~1.5 when switching the matrix for a distinct detergent; however, higher changes were also observed, e.g., differences of 2.4-fold and 4.2-fold for the CMC values of PS20 and Nereid, respectively, when comparing the recombinant protein matrix (14 °C) to ddH_2_O (25 °C). It should be noted, though, that the actual concentration of detergents as employed in S/D treatment in biotechnological manufacturing processes is considerably higher than the CMC values determined for any detergent–matrix combination in our study (e.g., ~50-fold higher for TX-100, which is utilized at 1% *w*/*w*, i.e., ~16 mM, in manufacturing of immunoglobulin [4]). Hence, potentially contaminating lipid-enveloped viruses would always be confronted with detergents in their micellar form.

In general, the effect of the surfactant structure on the resulting CMC is not very well documented in the literature. For instance, the CMC of nonionic surfactants has been observed to be lower compared to ionic surfactants. The CMC of surfactants mainly depends on the hydrophobicity of the amphiphiles i.e., the CMC decreases rapidly with increase in the alkyl chain length of the surfactant [11,18]. PS80, for instance, has a lipophilic chain that is more than twice as long as the lipophilic chain of TX-100 or Nereid, and therefore a much lower CMC. Furthermore, it is conceptually comprehensible that polysorbate surfactants, due to their multihydrophilic chains, will form the spherical structure of the micelles more easily and with fewer molecules than linear detergent surfactants such as TX-100 or Nereid. As a result, the packing of the hydrophobic tails forms the core, while the multi-hydrophilic heads are exposed outside and in contact with the aqueous environment, and may form the micelle sphere at a low surfactant concentration. In line with this, for all conditions investigated in our study, the CMCs of PS80 and PS20 were considerably lower than the CMCs of TX-100, Nereid and TX-100R. Yet, the latter detergents were shown to be more potent with respect to virus inactivation in previous studies [10], even though it is well accepted that polysorbate-based detergents have convenient features for biotechnological applications, i.e., good biocompatibility and low toxicity [19]. From a chemical point of view, this could be rationalized by the 3D geometric state of the detergents being linear and slender for the TX-100 family and more spherical for the polysorbate family (Figure 1). The bulkiness of the detergent having a sorbate core branched with several polyethylene glycol chains might prevent a smooth insertion in a virus’ lipid membrane, which is necessary for effective disruption. Higher concentrations of such a detergent or longer incubation times are therefore needed to achieve similar viral reduction. Micelle formation at a low concentration in this case would not correlate with the ease of membrane insertion/disruption. The hypothesis that the CMC might not always be a qualified indicator of antiviral potency is also supported by a study on detergent interaction with murine leukemia virus-like particles (VLPs); while TX-100 was highly effective in VLP membrane lysis, this was not the case for other detergents, e.g., PS20 and PS80, even when these were applied at concentrations way above their CMC [20].

Recently, the membrane-disrupting properties of TX-100 and another potential surrogates (Simulsol SL-11W) have been investigated, and it was proposed that the phospholipid membrane is only disrupted at or above the CMC [12]. Our results suggest that this might only be partially accurate; if TX-100, Nereid, or TX-100R were applied below their CMC, although their reduction factors were somewhat compromised compared to concentrations slightly above their CMC, virus inactivation was still evident. On the one hand, it cannot be ruled out that micelles potentially present at the very beginning of our virus inactivation runs (i.e., right after the addition of the micelle-containing detergent solution to the process intermediate) accounted for the observed BVDV inactivation during the very first moments of the experiments (before locally high detergent concentrations fell below the CMC due to intensive stirring). On the other hand, although the general understanding of the S/D mechanism is that formed micelles are the active molecular entities in the disruption mechanism of the viral lipid membrane, the presence of detergent micelles might not be an absolute prerequisite for virus inactivation. In line with this, at least for TX-100, the binding to the envelope of the Semliki Forest virus was shown to start below the CMC of this detergent [21].

In summary, the CMCs of detergents were determined in relevant biopharmaceutical product intermediates for the first time, revealing an overt dependency of this parameter on the matrix. At least for the detergents investigated, the propensity to form micelles might not correlate with antiviral potency. Finally, although virus inactivation below the CMC was less effective, our results challenge the indispensability of micelles for virus inactivation. Future studies should shed light on the question as to whether this phenomenon also translates to molecules other than the TX-100 family, e.g., alcohol ethoxylate or ionic detergents.

## 4. Materials and Methods

### 4.1. Detergents

The following detergents were commercially available: Triton X-100 (TX-100; Merck (Rahway, NJ, USA) Cat. No. 108643), Triton X-100 reduced (TX-100R; Sigma Aldrich (St. Louis, MO, USA), Cat. No. X100RS), Polysorbate 80 (PS80; Merck, Cat. No. 817061), and Polysorbate 20 (PS20; Merck, Cat. No. 44112). Nereid (4-(1,1,3,3-tetramethyl-butyl)benzyl-polyethylene glycol) is a proprietary compound, synthetized by Takeda as previously described [16]. Detergent stock solutions were prepared gravimetrically, i.e., by weighing the respective detergent on an analytical scale, followed by addition of the respective matrix to reach the targeted molar concentration of the detergent.

### 4.2. Matrices

The CMCs of detergents were determined in double-distilled water (ddH_2_O; Fresenius, Bad Homburg, Germany), and in two biotechnological process intermediates relevant to plasma-derived and cell-derived/recombinant products, respectively. The plasma protein matrix was fraction II, a process intermediate for the manufacturing of immunoglobulin upstream of the virus inactivation and removal steps. The matrix had a pH of 5.2 and was filtered through a 0.2 µm filter, and absorbance at 280–320 nm was adjusted to 28 AU/mL using a sodium chloride buffer. For the recombinant protein matrix (based on the composition of a protein A chromatography eluate from a monoclonal antibody production process), a glycine buffer (130 mM in ddH_2_O; pH 3.5) was combined with human albumin (25%, Baxter AG, Vienna Austria; final concentration 3.5 g/L), followed by pH adjustment to 5.1 with 500 mM sodium acetate (in ddH_2_O) and 0.2 µm filtration.

### 4.3. Force Tensiometry Measurements

CMC determinations were conducted with a force tensiometer (KRÜSS K100C, KRÜSS GmbH, Hamburg, Germany) and evaluated with ADVANCE software (v 1.11.0.15801). The measurements either employed a cylindrical glass vessel (50 mL measurement volume) or a conical aluminum vessel coated with PTFE (10 mL measurement volume). These measurement chambers, as well as the solutions employed during measurement, were tempered via a cryostat.

Measurements started with a solution without detergent (i.e., the matrix of interest) that was pipetted into the measurement chamber. A run consisted of approximately 40 repeat surface tension determinations, each with the following automated steps: (i) addition of an exact volume of a detergent stock solution (i.e., the respective detergent dissolved in the matrix of interest, via a microdispenser); (ii) stirring (using magnetic stirring bars) for 15 s; (iii) aspiration of the added volume (via a second microdispenser); and (iv) surface tension determination using a Wilhelmy plate. At the end of each run, the software provided with the instrument was used for subsequent analysis steps. The surface tension was plotted over the log-transformed detergent concentration, and two regression lines were set manually, corresponding to the two phases of (i) progressive surface tension decrease (i.e., before the CMC was reached), and (ii) constant surface tension (i.e., after the CMC was reached). The first regression line was set to capture most of the measuring points to approach a coefficient of determination (R^2^) ≥0.999, or the highest possible R^2^ with a minimum of five measuring points. As in the second phase, surface tension stays rather constant (i.e., slope close to zero), choosing a minimum R² for optimal establishment of the second regression line was not appropriate. Instead, the second regression line was set by linking five consecutive measuring points above the CMC, starting at the first or second measuring point above the CMC. Subsequently, the CMC was calculated automatically from the interception of the two regression lines. A representative CMC measurement trace is provided in the Supplementary Materials (Figure S1). For each detergent–matrix combination, the CMC was averaged from three independent measurements, except for PS20 and the plasma protein matrix, where the CMC was averaged from two independent measurements. Surface tension measurements of PS80 in the recombinant protein matrix yielded inconclusive raw data (instead of the two phases described above, a constant decrease in surface tension across a wide concentration range was observed), which precluded the determination of the CMC for this detergent–matrix combination.

### 4.4. Virus Propagation and Titration

MDBK cells (source: ATCC/CCL-22) were propagated in growth medium (GM; consisting of Dulbecco’s modified Eagle’s medium (DMEM; 4.5 g/L Glucose), 10% fetal calf serum, 2 mM L-Glutamine, 1 mM sodium pyruvate, 1x non-essential amino acids, 0.15% sodium bicarbonate, and 100 µg/mL gentamycin sulfate, and used to generate stocks of bovine viral diarrhea virus (BVDV; strain: Nadl; source: ATCC/VR-1422). BVDV is a model virus for Hepatitis C virus (HCV), for which the transmission to recipients of plasma-derived medicines has happened before the introduction of S/D treatment. Titration of BVDV infectivity was accomplished with a median tissue culture infectious dose (TCID_50_) assay and employed BT cells (source: ATCC/CRL-1390, propagated in GM and seeded for titration in the same medium, except that FCS was replaced by 10% horse serum). The assay design included serial half-log_10_ sample dilution with 8-fold replicates per dilution over a total of 12 dilution steps. After incubation of BT cells for 7 days (36 °C, 5% CO_2_, humidified atmosphere), cytopathic effects were evaluated by microscopical visual inspection. TCID_50_ titers were calculated according to the Poisson distribution and expressed as log_10_[TCID_50_/mL]. Virus reduction factors were calculated in accordance with the EU Committee for Proprietary Medicinal Products guidance [22]. If no viral infectivity was detected in successive samples up until the final sample, the volume of all successive negative samples was taken into account for calculation of the assay detection limit and the resulting virus reduction factor.

### 4.5. Virus Inactivation Experiments

Twenty-five mL of filtered and diluted plasma protein matrix (see above) was tempered to 17 °C ± 1 °C and continuously mixed by a magnetic stirrer. Spiking with BVDV was performed at a ratio of 1:31, i.e., 0.8 mL of virus stock solution were added. Within 1 to 2 min after spiking, two samples for virus titration were drawn; the spike control (SC) was titrated immediately, while the hold control (HC) was incubated in the same cooling circuit as the spiked matrix and titrated at the end of the experiment. The spiked matrix was weighed to determine the necessary amount of a detergent working solution (prepared by 1:20 dilution in ddH_2_O) to reach the desired final concentration. Addition of detergent working solution was accomplished using a Hamilton syringe. Samples for virus titration were drawn at 1 to 2 min, 10 ± 1 min, 30 ± 1 min, and 59 ± 1 min after detergent addition.

## Data Availability

Raw data for conducted experiments may be provided by the authors to readers upon request.

## Supplementary Materials

The following supporting information can be downloaded at: https://www.mdpi.com/article/10.3390/ijms24097920/s1, Figure S1: Representative force tensiometry measurement for Nereid in recombinant protein matrix.

## Abbreviations

BVDV	Bovine viral diarrhea virus
CMC	Critical micelle concentration
ddH_2_O	Deionized water
HC	Hold control
HCV	Hepatitis C virus
HIV	Human immunodeficiency virus
PS20	Polysorbate 20
PS80	Polysorbate 80
RF(s)	Reduction factor(s)
SC	Spike control
SD	Standard deviation
S/D	Solvent/detergent
TnBP	Tri-n-butyl-phosphate
TX-100	Triton X-100
TX-100R	Triton X-100 reduced
