# Supplementary material for: Detergent-Mediated Virus Inactivation in Biotechnological Matrices: More than Just CMC
Source: Int J Mol Sci. 2023 Apr 27;24(9):7920. doi: 10.3390/ijms24097920 (PMC10177830; doi:10.3390/ijms24097920)
Supplement: Supplementary file 1 [file ijms-24-07920-s001.zip › ijms-2331638-supplementary.pdf]

International Journal of *Molecular Sciences*

Supplementary Figure 1

to manuscript

Detergent-mediated virus inactivation in biotechnological matrices: More than just CMC

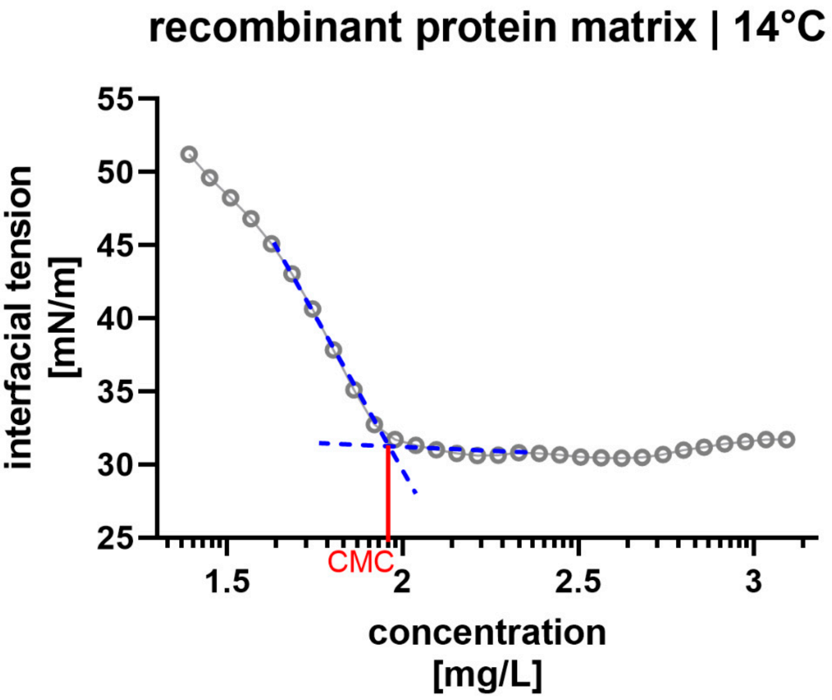

Figure S1. Representative force tensiometry measurement for Nereid in recombinant protein matrix. After addition of a defined amount of a detergent stock solution to the matrix, mixing and aspiration of the same volume that had been added before, the surface tension of the solution was determined. This procedure was repeated to obtain surface tension as a function of (increasing) detergent concentration; individual measurements are shown by grey circles. After the run, two regression lines (blue) corresponding to the two phases of (i) progressive surface tension decrease (i.e., before the CMC was reached), and (ii) constant surface tension (i.e., after the CMC was reached) were interpolated as described in *Section 4.3*. The CMC was subsequently calculated by the instrument software as the detergent concentration at which both regression lines intersect (red).
